# The influence of PSA autoantibodies in prostate cancer patients: a prospective clinical study-II

**DOI:** 10.18632/oncotarget.12620

**Published:** 2016-10-12

**Authors:** Kosei Nakajima, Lance K Heilbrun, Daryn Smith, Victor Hogan, Avraham Raz, Elisabeth Heath

**Affiliations:** ^1^ Department of Oncology, Karmanos Cancer Institute, School of Medicine, Wayne State University, Detroit, Michigan, USA; ^2^ Department of Pathology, Karmanos Cancer Institute, School of Medicine, Wayne State University, Detroit, Michigan, USA; ^3^ Biostatistics Core, Karmanos Cancer Institute, Wayne State University, Detroit, Michigan, USA

**Keywords:** PSA autoantibody, PSA test, false-results, prostate cancer, Galectin-3

## Abstract

The U.S. Preventive Services Task Force (USPSTF) has recommended against PSA-based screening for prostate cancer due to potential possibilities of false-results. Since no alternative test is available to replace it, we have initiated a trial with the purpose of establishing whether Galectin-3 (Gal-3) serum level and/or the patients immune response to PSA and Gal-3 antigens could complement the PSA test as diagnostic tools for prostate cancer patients. A blind, prospective, single institution, pilot study was conducted. A total of 95 men were recruited and classified into 5 different groups: healthy controls (Group1), newly diagnosed patients (Group2), no recurrence after local therapy (Group3), rising PSA after local therapy (Group4), and metastatic patients (Group5). The primary endpoints were the levels of serum PSA, PSA autoantibodies (AAPSA), Gal-3, and Gal-3 autoantibodies (AAGal-3). Data were analyzed by Spearmans rank correlation (rho) and least squares linear regression modeling. The expression levels of PSA, AAPSA, Gal-3, and AAGal-3 were determined in both healthy controls and prostate cancer patients. Negative correlations were observed between PSA and AAPSA levels among all 95 men combined (rho = −0.321, *P* = 0.0021; fitted slope −0.288, *P* = 0.0048), and in metastatic patients (rho = −0.472, *P* = 0.0413; fitted slope −1.145, *P* = 0.0061). We suggest an association between PSA and AAPSA, whereby the AAPSA may alter PSA levels. It provides a novel outlook for prostate cancer diagnosis, and should serve as a basis for an all-inclusive diagnostic trial centering on patients with metastasis.

## INTRODUCTION

In the framework of cancer diagnosis, biomarkers facilitate screening and detection of cancer, while monitoring the disease progression. Since the discovery of prostate-specific antigen (PSA), which possibly induces malignancy of prostate cancer, it has long been utilized for clinical diagnosis. PSA screening has contributed to earlier diagnosis and reduced incidence of metastatic disease. However, it also may often result in false-positive (higher PSA without cancer) or false-negative (lower PSA despite presence of cancer), leading to debates as to whether it should continue as a standardized screening method [1, 2].

Autoantibody (AA) is developed by the immune system in response to a self-antigen. In prostate cancer patients, several AAs were reported to react with cancer-related antigens, suggesting that such signatures could be used as the potential diagnostic biomarkers [3]. Specifically, autoantibody directed at PSA (AAPSA) has been identified in prostate cancer, benign hyperplasia, and prostatitis [4–6]. In this study, we hypothesized that AAPSA may affect the PSA level, which could result in an aberrant interpretation of the PSA assay.

As another cancer maker, the interest in Galectin-3 (Gal-3) stems from the evidence that it is a pro-inflammatory sugar-binding protein involved in prostate cancer malignancy, and is considered to be a promising therapeutic target [7, 8]. Previously, we have reported that increased serum levels of Gal-3 were associated with metastatic prostate cancer, inferring a possible complementary diagnostic marker to PSA [9, 10]. Similarly to AAPSA, cancer patients harbor autoantibody directed at Gal-3 (AAGal-3) [11, 12]. The generated AAPSA and AAGal-3 by activated immune system could directly prolong overall survival in prostate cancer patients [13].

The purposes of this study were 1- to determine the expression levels of AAPSA, Gal-3, and AAGal-3 as diagnostic accompaniments of the PSA test, and 2- to examine the relationship between PSA and AAPSA and between Gal-3 and AAGal-3 along with the clinical status of the patients enrolled.

## RESULTS

### Gal-3 and PSA autoantibodies are prevalent in men

A masked, prospective, single institution, pilot study was planned. A total of 95 participants was classified into 1 of 5 groups: healthy controls with no history of current invasive cancer (Group 1); newly diagnosed patients with intact prostate cancer (Group 2); patients who had no evidence of disease recurrence post local therapy (Group 3); patients with rising PSA after local therapy (Group 4); or patients with metastatic prostate cancer (Group 5). After patients’ serum samples were obtained, immunoblots were performed using recombinant human Gal-3 and PSA proteins. The results visualized the AA directed at Gal-3 and PSA (Figure S1). Next, in order to quantify the AA levels accurately, customized ELISA plates were established. All collected serum samples of 95 individuals were adequately available for the measurement. If men had a value under detection, *i.e*. PSA value of < 0.1 ng/ml or AA value of < 0.0048 μg/ml, numeric values of 0.05 ng/ml or 0.0024 μg/ml were assigned respectively to permit statistical analysis. Table 1 shows descriptive statistics of all 95 men. The mean Gal-3 was 14.74 ng/ml [90% CI: 13.85 - 15.62], the mean PSA was 17.14 ng/ml [4.31 - 29.96], the mean AAGal-3 was 12.73 μg/ml [11.17 - 14.28], and the mean AAPSA was 3.73 μg/ml [2.84 - 4.63]. These results indicate that AAPSA and AAGal-3 are prevalent in men.

Table 1AAPSA and AAGal-3 are prevalent in healthy controls and prostate cancer patients

**Table:** All 95 men

Variable	N	Median	Quartile Range	Mean	Std Dev	Minimum	Maximum	Lower 90% CL for Mean	Upper 90% CL for Mean
Gal-3 (ng/ml)	95	13.87	4.71	14.74	5.19	5.30	37.75	13.85	15.62
PSA (ng/ml)	95	1.90	7.05	17.14	75.23	0.05	694.50	4.31	29.96
AAGal-3 (ug/ml)	95	11.14	12.57	12.73	9.12	0.00	41.01	11.17	14.28
AAPSA (ug/ml)	95	1.44	5.74	3.73	5.25	0.00	25.62	2.84	4.63

**Table:** Group1: Healthy control

Variable	N	Median	Quartile Range	Mean	Std Dev	Minimum	Maximum	Lower 90%CL for Mean	Upper 90%CL for Mean
Gal-3 (ng/ml) PSA (ng/ml) AAGal-3 (ug/ml) AAPSA (ug/ml)	19 19 19 19	13.49 1.90 11.53 2.14	5.01 2.00 15.89 4.18	14.74 1.97 13.17 3.52	6.24 1.35 10.08 4.73	9.41 0.20 0.62 0.00	37.75 5.40 37.97 17.96	12.26 1.44 9.16 1.64	17.22 2.51 17.18 5.40

**Table:** Group2: Newly diagnosed

Variable	N	Median	Quartile Range	Mean	Std Dev	Minimum	Maximum	Lower 90%CL for Mean	Upper 90%CL for Mean
Gal-3 (ng/ml) PSA (ng/ml) AAGal-3 (ug/ml) AAPSA (ug/ml)	19 19 19 19	12.24 7.60 11.51 1.01	4.48 4.80 13.01 11.97	14.01 7.21 14.02 4.69	6.65 3.74 10.37 7.29	6.28 0.80 0.00 0.00	37.47 13.30 41.01 25.62	11.36 5.72 9.89 1.79	16.65 8.70 18.14 7.59

**Table:** Group3: No recurrence

Variable	N	Median	Quartile Range	Mean	Std Dev	Minimum	Maximum	Lower 90%CL for Mean	Upper 90%CL for Mean
Gal-3 (ng/ml) PSA (ng/ml) AAGal-3 (ug/ml) AAPSA (ug/ml)	19 19 19 19	13.69 0.05 16.84 5.74	4.61 0.00 16.90 7.37	15.05 0.05 15.99 5.26	5.33 0.00 9.11 5.15	9.71 0.05 0.00 0.00	33.54 0.05 30.76 15.99	12.93 . ^†^ 12.37 3.21	17.18 . ^†^ 19.62 7.31

**Table:** Group4: Rising PSA

Variable	N	Median	Quartile Range	Mean	Std Dev	Minimum	Maximum	Lower 90%CL for Mean	Upper 90%CL for Mean
Gal-3 (ng/ml) PSA (ng/ml) AAGal-3 (ug/ml) AAPSA (ug/ml)	19 19 19 19	14.30 1.60 11.14 0.67	5.37 5.60 7.52 3.16	14.61 12.15 10.59 1.54	3.23 27.20 5.28 1.67	10.09 0.30 0.00 0.00	20.84 93.90 19.81 5.53	13.32 1.33 8.49 0.88	15.89 22.97 12.69 2.21

**Table:** Group5: Metastasis

Variable	N	Median	Quartile Range	Mean	Std Dev	Minimum	Maximum	Lower 90%CL for Mean	Upper 90%CL for Mean
Gal-3 (ng/ml) PSA (ng/ml) AAGal-3 (ug/ml) AAPSA (ug/ml)	19 19 19 19	15.46 5.20 6.67 1.51	5.36 46.15 13.76 6.69	15.28 64.30 9.87 3.65	4.18 160.53 9.39 5.54	5.30 0.05 0.00 0.00	22.25 694.50 30.19 21.38	13.62 0.43 6.13 1.44	16.95 128.16 13.60 5.85

Descriptive statistics of the 4 measured variables in this study: Gal-3, PSA, AAGal-3, and AAPSA for all 95 men combined and for each clinical classification. † Due to the lack of any variation in PSA values for Group 3 (No recurrence), these confidence limits (CL) cannot be calculated.

**Figure 1:**
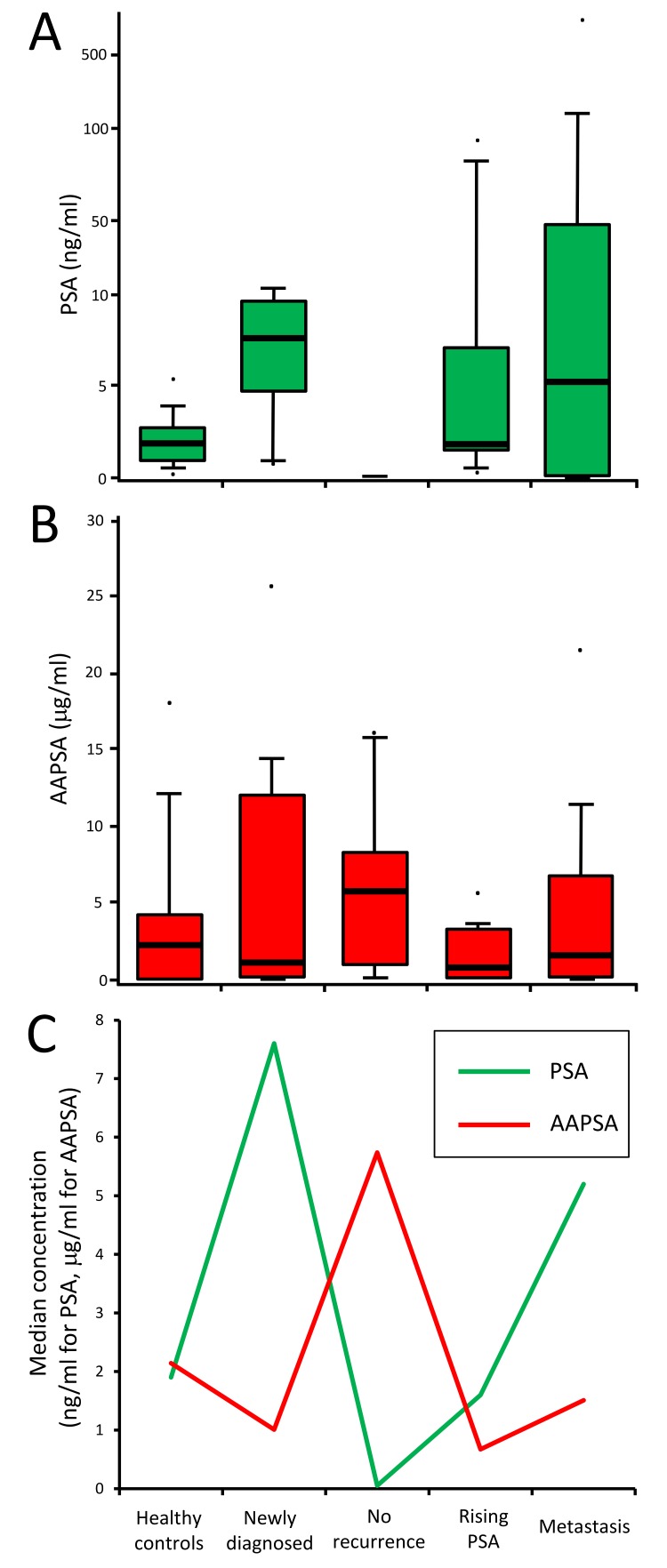
Associations between PSA and AAPSA: possible reverse transitions **A**.-**B**. Box plots show value distributions of **A.** PSA, and **B.** AAPSA by clinical group. For the PSA graph, a log_10_ scale was used on the Y-axis to accommodate some extreme values. Whisker heights indicate the 90th and the 10th percentiles of the distribution. Bold horizontal lines within the box indicate the median values. The dots indicate maximum or minimum values of each group. **C**. The median values of PSA and AAPSA were plotted as a line graph. The green line indicates a transition of PSA level. The red line indicates a transition of AAPSA level. An opposite transition between PSA and AAPSA was noted across the 5 clinical classifications.

### Possible associations between autoantibodies and antigen levels of Gal-3 and PSA

To find out the possible clinical significance(s) of the prevalent AAs, we next analyzed the 4 variables (Gal-3, AAGal-3, PSA, and AAPSA) along with the patients’ classifications. The median Gal-3 levels of each group were (Group1: healthy controls) 13.49 ng/ml, (Group 2: newly diagnosed) 12.24 ng/ml, (Group 3: no recurrence) 13.69 ng/ml, (Group 4: rising PSA) 14.30 ng/ml, and (Group 5: metastasis) 15.46 ng/ml, respectively (Figure S2A). There was no significant difference in the Gal-3 levels across the five clinical groups (*P* = 0.3524). Next, AAGal-3 levels were analyzed, and the median AAGal-3 levels of each group were (Group1: healthy controls) 11.53 μg/ml, (Group 2: newly diagnosed) 11.51 μg/ml, (Group 3: no recurrence) 16.84 μg/ml, (Group 4: rising PSA) 11.14 μg/ml, and (Group 5: metastasis) 6.67 μg/ml (Figure S2B). The patterns of the median values of Gal-3 and AAGal-3 were displayed graphically (Figure S2C).

**Figure 2:**
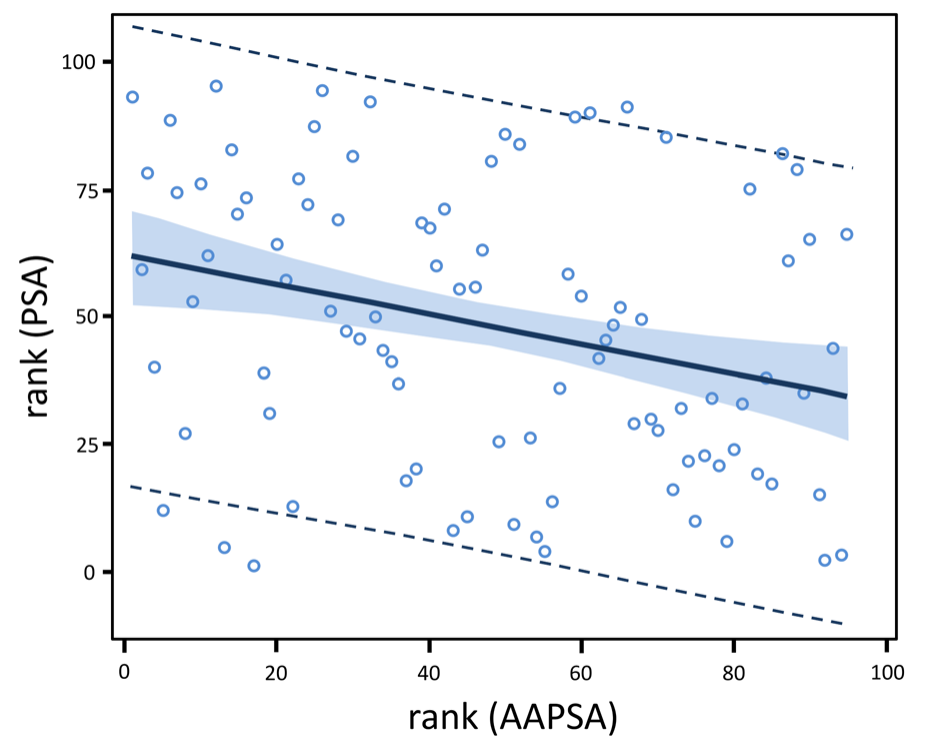
AAPSA reduces the level of serum PSA concentrations in men The linear regression model fit plot shows a negative association between PSA and AAPSA. The lines at the outer edges of the blue band define the 90% confidence limits for the mean of rank (PSA) for a given value of rank (AAPSA). The dashed lines define the 90% prediction limits for an individual value of rank (PSA) for a given value of rank (AAPSA). The fitted regression model for all 95 men combined is: rank (PSA ng/ml) = 61.812 - 0.288*rank (AAPSA μg/ml).

The possible association between PSA and AAPSA was evaluated next. The median PSA values of each group were (Group1: healthy controls) 1.90 ng/ml, (Group 2: newly diagnosed) 7.60 ng/ml, (Group 3: no recurrence) 0.05 ng/ml, (Group 4: rising PSA) 1.60 ng/ml, and (Group 5: metastasis) 5.20 ng/ml (Figure 1A). The median AAPSA values of each group were (Group1: healthy controls) 2.14 μg/ml, (Group 2: newly diagnosed) 1.01 μg/ml, (Group 3: no recurrence) 5.74 μg/ml, (Group 4: rising PSA) 0.67 μg/ml, and (Group 5: metastasis) 1.51 μg/ml (Figure 1B). Of note, an overlay of the median PSA and median AAPSA levels revealed reverse transitions for Groups 2-5. The median PSA level showed a pattern of ‘High-Low-High-High’, whereas the median AAPSA level presented a ‘Low-High-Low-Low’ pattern (Figure 1C), implying that AAPSA might yield an underestimate of the PSA level.

### AAPSA levels are negatively associated with PSA concentration

The above prompted the statistical evaluation of whether higher AAPSA levels are associated with lower PSA level or *vice versa.* Table 2 summarizes the Spearman correlation coefficients. Since Group 3 (no recurrence) had same PSA value near zero (0.05 ng/ml) with no variation, it was not amenable to statistical analysis. The results showed that all 5 rho values were negative; 2 of them were statistically significantly different from zero, *i.e*. rho = −0.312, *P*-value = 0.0021 among all 95 men, and rho = −0.472, *P*-value = 0.0413 in metastatic patients, suggesting that AAPSA was negatively associated with PSA level.

**Table 2:** AAPSA levels are negatively associated with PSA concentration

Group	rho	*P*-value	Slope	90% CI	*P*-value
All men	−0.312	0.0021 *	−0.288	( −0.453, −0.122 )	0.0048 *
Healthy controls	−0.255	0.2929	−0.076	( −0.302, 0.150 )	0.5676
Newly diagnosed	−0.200	0.4118	−0.141	( −0.313, 0.031 )	0.1733
No recurrence	NA	NA	NA	NA	NA
Rising PSA	−0.026	0.9155	0.399	( −2.792, 3.591 )	0.8303
Metastasis	−0.472	0.0413 *	−1.145	( −1.783, −0.508 )	0.0061 *

The table shows Spearman rank correlation coefficients (rho values) and their P-values for all 95 men combined and separately for each clinical group (left). The fitted slope, its 90% confidence interval (CI), and P-value are also shown for the linear regression model of transformed PSA and transformed AAPSA (right). NA, not amenable to analysis since the 19 men in Group3 (no recurrence) all had identical PSA values of 0.05 ng/ml. A P-value of less than 0.05 was considered statistically significant (*).

Further, we performed linear regression modeling of PSA and AAPSA, and visually examined the relationship. Only the ranks transform Normalized both variables among all 95 men combined. Various transforms were needed for the individual clinical groups. Gal-3 was included as a covariate in bivariate models. On the other hand, AAGal-3 was not included in the analysis of relationship PSA and AAPSA because in a general biological understanding, antigen-antibody reaction is considered to be a specific interaction, likewise AAGal-3 affects neither the level of PSA nor AAPSA.

For all 95 men combined, the fitted slope (−0.288) was negative, and statistically significant (*P* = 0.0048) (Figure 2). Including rank (Gal-3) as a covariate resulted in only a negligible change in the estimated slope (−0.298) and its *P*-value (0.0041). Excluding the 1 leverage point in the bivariate model resulted in negligible changes in the estimated slope (−0.283) and its statistical significance (*P* = 0.0079). Thus, the covariate adjustment and the sensitivity analysis suggested a robust and negative relationship of rank (PSA) with rank (AAPSA).

For Groups 1 (healthy controls), Group 2 (newly diagnosed), and Group 4 (rising PSA), no significant association of transformed PSA and transformed AAPSA was identified from the linear regression models. Neither Gal-3 covariate adjustment nor exclusion of model leverage points changed those 3 group-specific conclusions (data not shown). Group 3 (no recurrence) was not amenable to regression modeling at all, since all 19 men had PSA values uniformly near zero (0.05 ng/ml).

For Group 5 (metastatic prostate cancer), the fitted slope (−1.145) was negative, and statistically significant (*P* = 0.0061). Including ln (Gal-3) as a covariate did not appreciably change the estimated model statistics. For the regression models, either 1 or 2 leverage points were identified, which were referred to Model 1 and Model 2, respectively. Exclusion of those leverage points weakened the statistical significance of the estimated slope of each of the models. For Model 1, the new slope estimate was −1.054 (very small change) with *P* = 0.0548. For Model 2, the new slope estimate was −1.089 (very small change) with *P* = 0.0498. Hence, exclusion of the leverage points still revealed a moderately significant negative relationship between ln (PSA) and sqrt (AAPSA) in Group 5.

With respect to the association Gal-3 and AAGal-3, we did not observe a statistically significant positive/negative association either in all 95 men combined or in any specific clinical group (Data not shown).

## DISCUSSION

In the present study, we suggest that prevalent AAPSA in men may reduce the level of serum PSA concentrations, possibly due to immuno-precipitation, which might contribute to false-negative results. On the other hand, lower AAPSA levels may allow for an increase in PSA level, which could lead to false-positive results. The relationship between PSA and AAPSA should be further explored for biological and mechanistic insight. In our study, the PSA reacted with AAPSA can be represented by a linear regression model: rank (PSA ng/ml) = 61.812 - 0.288*rank (AAPSA μg/ml). A potentially reduced PSA level should be taken into consideration when viewing PSA test results. The small number of samples per clinical group in the current study precludes confirming causality between the presence/level of AAPSA and false-results. A larger investigation is needed to ascertain whether a diagnostic criterion including AAPSA might improve erroneous results from PSA testing, and the same idea would be applicable to other cancer-inducible antigens such as Gal-3.

As another study limitation, we did not follow the patient's status and were unable to observe a correlation between AAPSA and the clinical consequence of prostate cancer progression due to the nature of cross sectional study. Contemplating the above association that AAPSA reduces PSA, and PSA is involved in the malignancy of prostate cancer [2], it is possible to assume that AAPSA suppresses, at least in part, the progression of prostate cancer in patients.

In conclusion, altered PSA level of expression by AAPSA may affect the PSA test accuracy. We hypothesize that anti-PSA antibodies should be considered as a novel monitoring element for prostate cancer status.

## MATERIALS AND METHODS

### Study patients

Eligible men were age ≥ 18 (if already diagnosed with prostate cancer). Previous history of chemotherapy may possibly confound AA levels because in general, the treatment suppresses the immune function. Such patients were not included in this study. To classify prostate cancer patients, they were examined by PSA, trans-rectal ultrasound (TRUS), and prostatic biopsy. Metastatic lesions were detected by chest X-rays, CT, MRI, bone scan, and/or F-18 sodium fluoride positron emission tomography (NaF-PET). From October 2013 to July 2015, patients were recruited from genitourinary oncology clinics, Karmanos Cancer Institute, and then gave informed consent to be participants. The study-related information of patients was recorded in the Online Collaborative Research Environment (OnCore^®^) database. Patients’ whole bloods were collected using two 5 ml serum separator tubes, and were centrifuged at 5000 rpm for 5 minutes to separate serum from cellular components. The serums were aseptically transferred to cryovials labeled with limited information in a safety cabinet, and then frozen at −80˚C. Due to the need for unbiased assays, the clinical information (*i.e*., patient Group identification) was masked to the laboratory investigators.

### ELISA

Customized ELISA plates were generated to detect AAGal-3 and AAPSA contained in prostate patients’ sera. First, human recombinant Gal-3 [14] or PSA (Novus Biologicals, CO) were diluted by 100nM bicarbonate/carbonate coating buffer (pH9.6). Then, 94ng of recombinant Gal-3 and 10ng of recombinant PSA were incubated in each well of Nunc-immuno™ MicroWell 96 well solid plates (Thermo scientific, Waltham, MA) for 1 hour at 37 °C. Simultaneously, human normal IgG (Invitrogen, Carlsbad, CA) was serially diluted and incubated on the plate for the standard curve. After fixation of the proteins, the liquids were discarded. The wells were washed 4-times using TBS with tween 20 (0.1%). Blocking was performed using 1% BSA/coating buffer for 1 hour at 37°C. Patient's sera were diluted 80-fold using phosphate buffered saline (PBS) with 0.75% BSA plus 0.1% tween 20 for AAPSA detection. For AAGal-3 detection, the sera were diluted 160-fold using PBS with 1% BSA plus 0.5% tween 20. Then, 100ul of diluted sera were incubated for 1 hour at 37°C. After washing, anti-human IgG peroxidase-conjugated antibodies (Rockland, PA) were reacted for 1 hour at 37°C. Then, tetra-methyl-benzidine (TMB), a substrate for peroxidase, was incubated for 20 min at room temperature. The enzymatic reactions were terminated by addition of 0.5M sulfonic acid. Absorbance was measured at 450nm. In order to eliminate non-specific reactions, wells without recombinant proteins were also prepared, and incubated with each patient's serum. The net absorbance was calculated as following formula: (absorbance with recombinant protein) - (absorbance without recombinant protein). Then, concentration was determined by extrapolation into the standard curve, whereby the range of 4.8 - 312.5 ng/ml was measurable. As for Gal-3 concentration, Galectin-3 ELISA kit (BG Medicine, Waltham, MA) was used. The measurements were also performed in duplicate following the manufacturer’s. protocol. The mean of the duplicates was used in all statistical analyses. An ELISA plate stratified randomization procedure was used to assign patients’ samples to wells for each plate so as to minimize confounding due to plate effects, row effects, or column effects.

### Statistical methods

#### Design

The objective was to identify Gal-3, AAGal-3, PSA, and AAPSA in the serum of men in 5 different states of prostate cancer. The primary statistical endpoints were the levels of each of those 4 study biomarkers. Within each group of men, it was desired to estimate the mean biomarker level to within 0.40 standard deviations (SD’s) of the true mean, with 90% confidence. The study required *N* = 19 men per group, hence 19*5 = 95 patients in total. The required sample size per group was determined *via* the ‘Confidence Intervals for One Mean’ program in the Power And Sample Size (PASS) 11 software [15].

#### Analysis

For all 95 men, and separately for each group, Gal-3, PSA, and their AA data were summarized with standard descriptive statistics, number of each group (N), mean, standard deviation (SD), median, interquartile range (IQR), minimum value, maximum value, and the 90% confidence interval (CI) for the mean. Statistical graphics (boxplots and line plots) of PSA, Gal-3, and their AA data were also generated for each group. The nonparametric Kruskal-Wallis test was used to compare a given biomarker across clinical groups. To first evaluate the association between any pair of continuous variables, the nonparametric Spearman's rank correlation coefficient was calculated to obtain a provisional indication of the direction and strength of linear association. To characterize the statistical relationship between Gal-3 and AAGal-3 or between PSA and AAPSA, ordinary least squares (OLS) linear regression modeling was used. Normality testing of all 4 study variables was performed separately within each of the 5 clinical groups, and for all 95 men combined. Ten transformations were generated (null [no transform], ln, log_10_, square root, cube root, fourth root, fifth root, inverse, inverse squared, and rank), and tested for Normality. Four tests of Normality were performed: Shapiro-Wilk, Kolmogorov-Smirnov, Cramer-vonMises, and Anderson-Darling. Non-Normality was concluded if at least 2 of those 4 tests were significant at the 0.01 alpha levels. If more than 1 transform Normalized a given study variable, the transform that was the mathematically simplest was chosen. Then, linear regression modeling was performed using transformed variables. Model residuals were thoroughly examined to assess goodness of fit. Sensitivity analyses were also conducted after excluding leverage points identified in the regression models. The SAS software version 9.4 (SAS Institute, Cary, NC) were used for statistical analyses. All tests of statistical significance were two-sided. A *P* value of less than 0.05 was considered statistically significant. Given the pilot nature of the study, no adjustments were made for multiple comparisons.

Other methods are described in the Supplementary Methods.

## SUPPLEMENTARY MATERIALS FIGURES AND TABLES


