# Supplementary material for: The influence of PSA autoantibodies in prostate cancer patients: a prospective clinical study-II
Source: Oncotarget. 2016 Oct 12;8(11):17643–50. doi: 10.18632/oncotarget.12620 (PMC5392275; doi:10.18632/oncotarget.12620)
Supplement: Supplementary file 1 [file oncotarget-08-17643-s001.pdf]

The influence of PSA autoantibodies in prostate cancer patients: a prospective clinical study-II

Supplementary Methods

Immunoblot

In order to directly visualize AA contained in a patient's serum, immunoblot was performed. Human recombinant Gal-3 or human recombinant PSA (R&D systems, Minneapolis, MN) were mixed with sample buffer and 500ng of recombinant Gal-3 and 100ng of recombinant PSA were subjected to 10% SDS-polyacrylamide gel electrophoresis (PAGE) in non-reducing conditions. The transferred membrane was quenched with 0.1% casein / tris-buffered saline (TBS) for 1 h, and then was cut between the lanes. The strips were incubated separately with 60-folds diluted patient serums for overnight, and then incubated with anti-human IgG secondary antibody conjugated with Dylight™ 680 (Rockland, Gilbertsville, PA) for 1 h. As positive controls, anti-Gal-3 rat monoclonal antibody (TIB166, ATCC) and anti-PSA goat polyclonal antibody (C-19, Santa Cruz Biotechnology) were used. Reacted AA with the recombinant proteins on the strips were visualized by Odyssey Infrared Imaging System and Odyssey application software (LI-COR Biosciences, Lincoln, NE).

Fig. S1

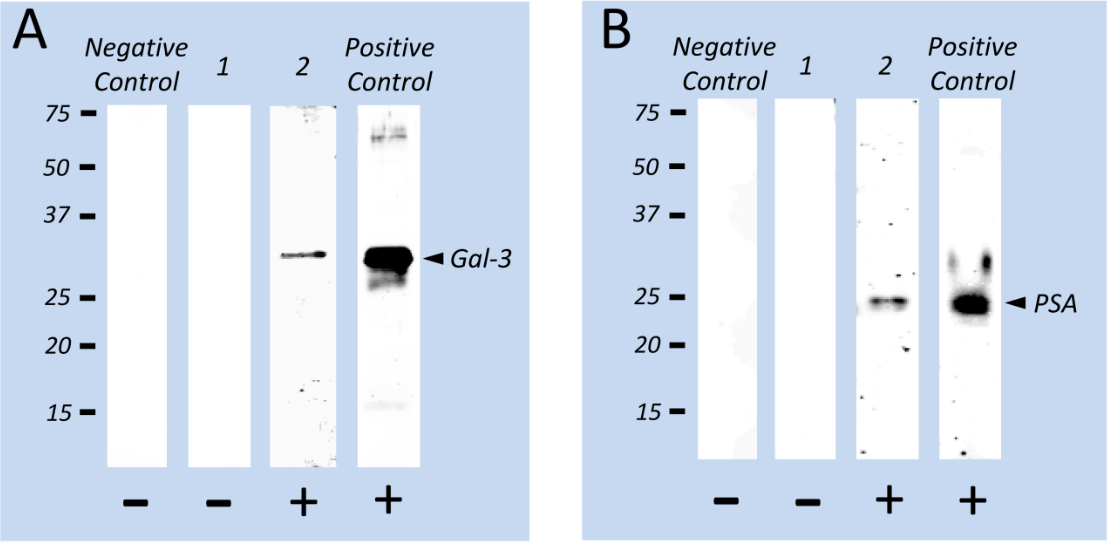

Figure S1. **Visualization of autoantibody directed at Gal-3 and PSA in patients' serum.** For negative control, the strip was stained with no serum. On lanes No.1-2, patients' serums were used for staining. For positive control, the strips were stained with anti-Gal-3 antibody or anti-PSA antibody. **A.** AAGal-3 was detected on patient No.2. **B.** AAPSA was detected on patient No.2.

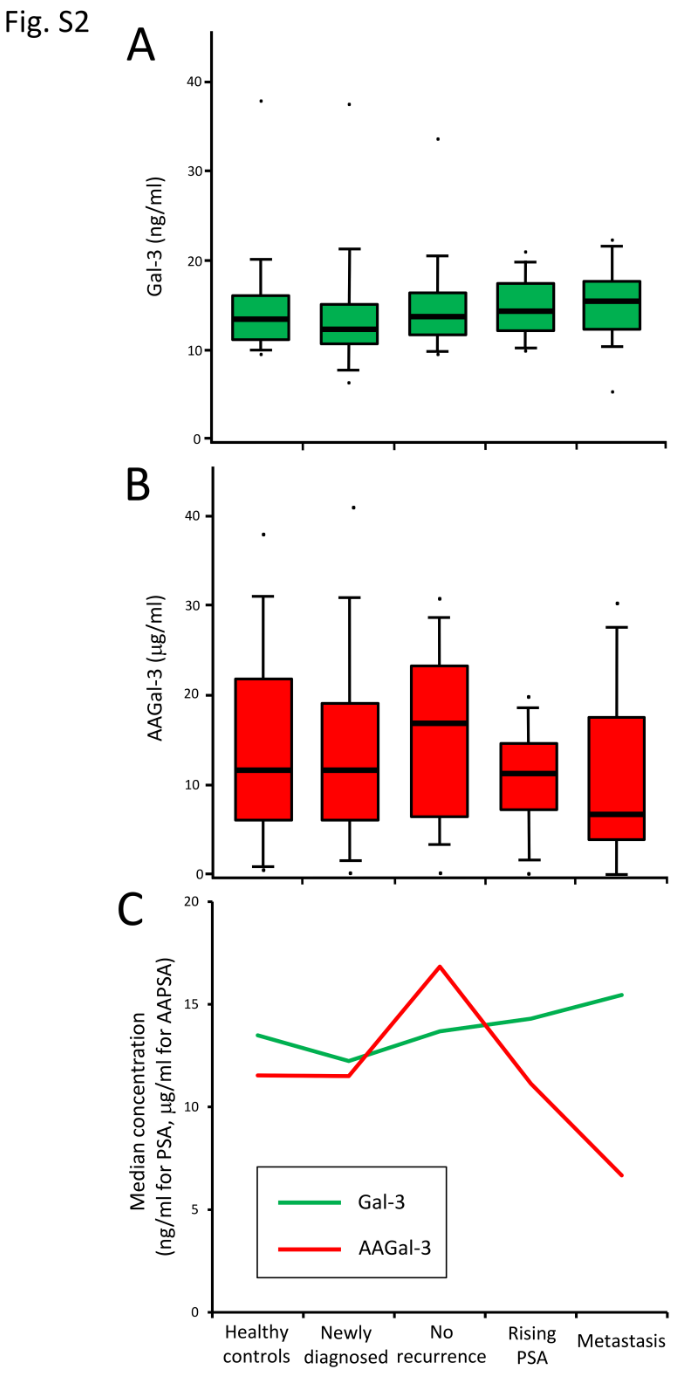

Figure S2. Associations between Gal-3 and AAGal-3.

A-B. Box plots show statistics of the distributions of Gal-3 (**A**), and AAGal-3 (**B**) by clinical group. Whisker heights indicate the 90th and 10th percentiles of the distribution. Bold horizontal lines within the box indicate the median values. The dots indicate maximum or minimum values of each group. **C**. The median values of Gal-3 and AAGal-3 were plotted as a line graph across the 5 clinical groups. The green line indicates a transition of Gal-3 level. The red line indicates a transition of AAGal-3 level.
